# Hydrophobic Wafer-Scale High-Reproducibility SERS Sensor Based on Silicon Nanorods Arrays Decorated with Au Nanoparticles for Pesticide Residue Detection

**DOI:** 10.3390/bios12050273

**Published:** 2022-04-26

**Authors:** Fanhong Chen, Yupeng Zhao, Shaoxun Zhang, Shuhua Wei, Anjie Ming, Changhui Mao

**Affiliations:** 1 State Key Laboratory of Advanced Materials for Smart Sensing, GRINM Group Corporation Limited, Beijing 100088, China; chenfanhong@grinm.com (F.C.); 17863137556@163.com (S.Z.); 2 Department of Advanced Electronic Materials, GRIMAT Engineering Institute Co., Ltd., Beijing 101407, China; zhaoyp@mail.ncut.edu.cn; 3 School of Information Science and Technology, North China University of Technology, Beijing 100144, China; weishuhua@ncut.edu.cn

**Keywords:** SERS, hydrophobic, wafer-scale, reproducibility, malachite green

## Abstract

High sensitivity and reproducibility are highly desirable to a SERS sensor in diverse detection applications. Moreover, it is a great challenge to determine how to promote the target molecules to be more concentrated on the hotspots of the SERS substrate by engineering a surface with switching interfacial wettability. Along these lines, wafer-scale uniformly hydrophobic silicon nanorods arrays (SiNRs) decorated with Au nanoparticles were designed as the SERS substrate. Typically, the SERS substrate was fabricated by enforcing the polystyrene (PS) sphere self-assembly, as well as the plasma etching and the magnetron sputtering techniques. Consequently, the SERS substrate was treated by soaking within a n-dodecyl mercaptan (NDM) solution at different times in order to obtain adjustable wettabilities. By leveraging the electromagnetic enhancement resulted from the Au nanostructures and enrichment effect induced by the hydrophobicity, the SERS substrate is endowed with efficient SERS capabilities. During the detection of malachite green (MG), an ultralow relative standard deviation (RSD) 4.04–6.14% is achieved and the characteristic signal of 1172 cm^−1^ can be detected as low as 1 ng/mL. The proposed SiNRs’ structure presents outstanding SERS activity with sensitivity and reproducibility rendering thus an ideal candidate for potential application in analytical detection fields.

## 1. Introduction

Trace detection arouses great attention in the chemical and biological analysis for various disciplines including biomedicine [1,2], early diagnosis [3,4,5], environmental monitoring [6,7], and food safety [8,9], et al. The SERS is regarded as a quite promising technique for sensing trace substances and obtaining molecule structure information within the field of analytics due to high sensitivity and label-free characteristics [10,11,12]. The main contribution of the SERS is ascribed to the electromagnetic effect, which usually occurs by the existence of localized surface plasmons of the noble metal structure under the enforcement of laser irradiation [13,14]. In order to achieve a strong Raman scattering signal of the analyte material, considerable research efforts have been devoted to the fabrication of highly sensitive and versatile SERS metallic nanostructures [15,16,17,18], such as Au@Ag core-shell [19], Ag nanosheet [20], and CuO@Ag nanowire arrays [21], et al. On top of that, electron beam lithography [22], nanoimprinting [23], ion-beam sputtering [24], and chemical synthesis [25] techniques have been also frequently used during the assembly processes of the SERS micro-nanostructure. For instance, Tian and co-workers [26] constructed a CO molecules detection platform composed of a Au@Pt core-shell nanoparticles’ structure by enforcing seed-mediated growth methods. Interestingly, the SERS signal of the Au@Pt nanoparticles was about 200 times stronger than that of the roughened Pt electrodes and about 40 times higher than the employment of 12 nm Pt nanocubes. Additionally, Lei and co-workers [27] designed and prepared a cavity-based particle-in-quasi cavity architecture composed of hierarchical ZnO/Ag nanosheets and nanoprotrusions. The ZnO/Ag-based structures exhibited excellent in situ SERS detectability, with a detection limit as small as 10^−18^ M for R6G and 12.8% of the signal RSD value. These SERS structures possess outstanding signal amplification ability in conjunction with low detection limit, while some have anti-interference property, thus achieving real-time monitoring for environmental hazards. We have also to underline that the involved nanofabrication methods are facile and cost-effective. Based on previous works, our group have prepared a SERS substrate through assembling ordered hexagonal-packed PS nano-spheres arrays decorated with Au nanoparticles and clusters. The SERS substrate exhibited good detection sensitivity and the minimum limit for MG molecules reached 50 ng/mL [28].

The surface wettability technique offers new and unique possibilities in a wide range of applications such as liquid transportation [29], catalyst interface modification and gas diffusion layer design [30,31], as well as the distribution of bio-samples for diagnosis [32]. Initially, the contact state of the liquid droplet on the wettability substrate is greatly different. As a result, the hydrophilic interface has the ability to quickly induce the solution spreading. Vice versa, the liquid droplet on the hydrophobic interface can retain a more regular spherical shape. Consequently, the different properties of the hydrophilic and the hydrophobic interface result in diverse SERS performance by controlling the concentrating analyte molecules at hotspots [33,34,35]. Therefore, the interface wettability modification cannot be neglected and is considered as a key in order to effectively improve the SERS activity. The underlying idea is that an increased analyte concentration is enriched at the smaller area by tuning the wetting behaviors of the analyte droplet on the SERS substrates. In order to attain this goal, Song [36] proposed the fabrication of an Au-areoles array, which was deposited on a patterned superhydrophilic-superhydrophobic substrate by employing a selectively electrochemical method. The produced SERS microchip demonstrated a high-throughput detection of multiple analytes without any interference capabilities, and by localizing the probe molecules of R6G at the hotspots, it achieved a limit detection of 10^−15^ M. In analogy to the reasonable design on the plasmonic nanostructures, a comprehensive aqueous solution manipulation on the SERS surfaces with modulated wettabilities is also highly desirable, while in contrast to the intensive research, the later one remains rather vague due to the lack of fundamental understanding of advantages in the by-modulated wettabilities.

**Scheme 1:**
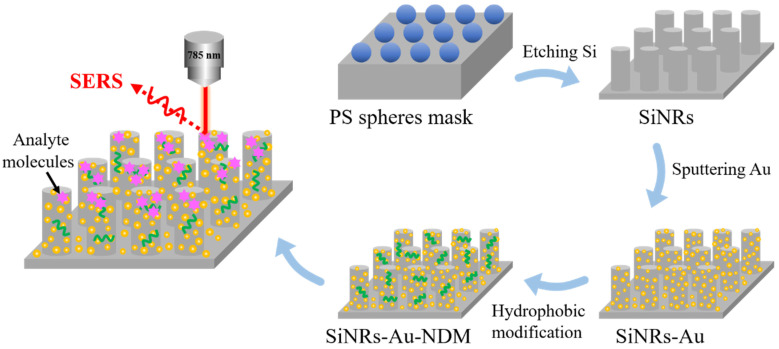
Schematic diagram of the fabrication processes of the hydrophobic SERS substrate.

Herein, in order to obtain a cost-effective, large-size and high-reproducibility SERS substrate with enforcing facile and controllable preparation procedures, we designed an ordered SiNRs’ structure decorated with Au nanoparticles. Moreover, the FDTD method was used for calculating and visualizing the electromagnetic field distribution of the coated Au nanoparticles SiNRs’ structure. Then, as it is illustrated in Scheme 1, a SiNRs’ structure with an average diameter of ~250 nm, a gap of ~50 nm and a length of ~700 nm was fabricated by applying the self-assembly and plasma etching methods, whereas ~20 nm Au nanoparticles were coupled by the magnetron sputtering deposition technique, which were integrated into a strong plasmonic SERS substrate. In the meantime, the SERS substrate was treated by soaking within NDM solution with different processing times in order to induce adjustable wettability. Finally, the sensitivity and reproducibility of the SERS substrate were confirmed through the detection of the pesticide residue of MG. This system could be potentially applied as an on-the-spot chemical detection element, as well as point-of-care medical diagnostics.

## 2. Materials and Methods

### 2.1. Preparation and Characterization of the SERS Substrate

All the employed chemicals were of analytical grade and used without enforcing any further purification. The close-packed PS self-assembled monolayer was prepared by following a process that was reported in our previous work [37]. In a typical procedure, firstly, the silicon wafer with 100 nm thickness of SiO_2_ was cleaned with acetone, ethanol and deionized water with the assistance of ultrasonication for several minutes. Then, it was placed on the homemade apparatus and filling deionized water. Subsequent, 6 mL Sodium dodecyl sulfate (SDS) were added in a concentration of 20 mM and the PS suspensions were mixed with 66% ethanol to the air/water interface through the tilted glass slide in the area limited by the ring and let stand for a period of 10 min and 1 h, respectively. Finally, the self-assembled monolayer was transferred into the Si wafer by letting the water flow out of the apparatus.

The preparation procedure of the ordered SiNRs decorated with Au nanoparticles is described as follows. Firstly, the diameter of the prepared close-packed PS spheres was reduced to the ordered required size by enforcing the inductively coupled plasma etching (ICP-98A, China) technique with O_2_ gas, while the gas flow rate was 900 sccm, the radio frequency (RF) was 800 W and the reaction time was 80 s as a nano-sized mask. Then, the SiO_2_ nanorod arrays were fabricated by a reactive ion (RIE-501, China) with CF_4_ gas. More specifically, the gas flow rate was 40 sccm, the RF power was 300 W, the total pressure was 1 Pa, and the etching time was 180 s for 10 times. Afterward, SiNRs were formed by RIE with O_2_ and SF_6_ gas, where the gas flow rate was 10 sccm and 60 sccm, respectively, the RF power was 300 W, the pressure was 5 Pa and the etching time was 420 s. The introduction of SiO_2_ as a dielectric layer can overcome the poor corrosion resistance of the PS sphere and improve the aspect ratio of SiNRs. In addition, the time-sharing etching was taken in order to minimize the structure melting and linking mechanism caused by the energy emitted during the etching processes. Finally, 20 nm of Au nanoparticles were deposited on the SiNRs arrays by the magnetron sputtering (QAM-4D, Japan) method with a pressure of 1.5 Pa, Ar flow rate of 50 sccm, power of 20 W and time of 20 s. The morphology of the PS monolayer, SiO_2_ and SiNRs nanorod arrays, as well as the Au nanoparticles, was characterized by carrying out scanning electron microscope (SEM, S-5500, HITACHI, Japan) and transmission electron microscope (TEM, Thermo Fisher Scientific Talos F200X) measurements.

Afterward, the prepared SERS substrates were immersed within an 0.01 mM NDM solution for about 60 s, 300 s and 900 s in order to acquire different wettability properties. The wettability characteristics of the SERS substrates were assessed by measuring the droplet contact angle by using the Dataphysics OCA20 system.

### 2.2. FDTD Simulation

The FDTD simulations were performed based on the SERS substrate models. The structures were illuminated with a plane wave directed along the *z*-axis. The employed periodic boundary conditions used a perfectly matched layer (PML) in order to adsorb the boundary conditions of the cell. The simulation time was set to 300 fs. Moreover, the dielectric constant for Au was selected as Johnson and Christy (the type of Au) from the database, while the refractive index of the underlying SiNRs was set to a value of 1.55, and the surrounding medium of air was 1.0.

### 2.3. Raman Analysis of the SERS Substrate

The SERS spectroscopy measurements were carried out on a laser microscope Raman (ATR8300, OPTOSKY, Xiamen, China). The employed excitation wavelength was 785 nm with an effective power of 20 mW, whereas a 20× objective was used during the detection, and the integral time was 10 s for two accumulations. In order to analyze the SERS performance, 6 µL of MG solution with different concentrations was dropped onto the SERS substrate surface and dried under ambient conditions. Subsequently, the SERS spectra of the analyte were recorded.

## 3. Results and Discussion

**Figure 1:**
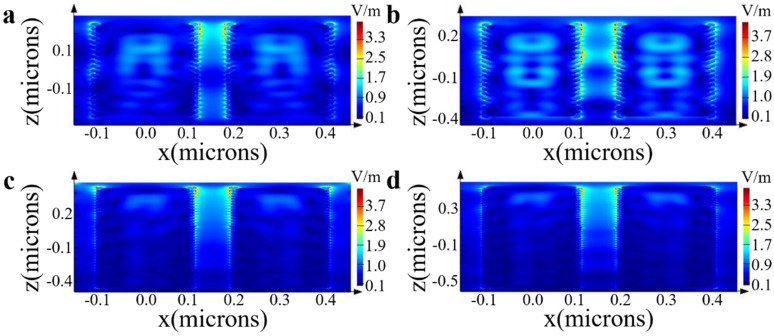
Distribution of the FDTD−simulated near−field electrical intensity in the wavelength of 785 nm with different SiNRs’ length. (**a**) 500 nm, (**b**) 700 nm, (**c**) 900 nm and (**d**) 1100 nm.

Since the local enhancement of the electromagnetic field in the SERS effect is considered the driving force, the calculation of the electric field distribution is quite critical for the successful design and synthesis of the SERS substrate structure. Figure S1 depicts the FDTD simulation model of the electric field enhancement effect at an excitation wavelength of 785 nm by irradiating the different structures from the upper side. As can be ascertained from Figure 1a–d, the electric field intensity does not enhance proportionally as the length of the SiNRs increases from 500 nm–1100 nm, while the highest electric field intensity at a length of 700 nm is achieved. By considering the above observations, although longer SiNR structures provide space for incorporating an elevated concentration of Au nanoparticles and as a result create significant hotspots, the masking impact of elongated SiNRs on the Raman signal is not negligible. Herein, noble-metal Au is selected as hotspot material due to its larger energy gaps of d and s electrons than those of transition metals, which are more likely prone to interband transitions.

**Figure 2:**
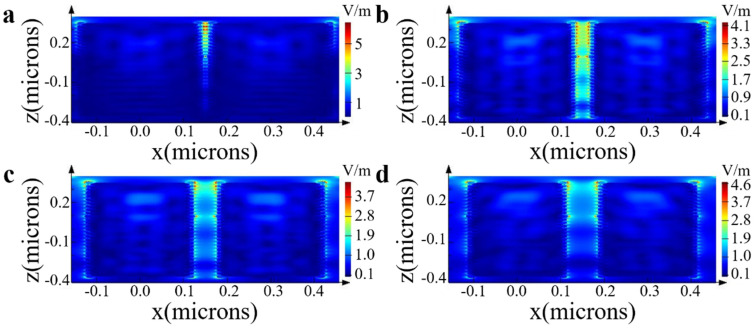
Distribution of the FDTD−simulated near−field electrical intensity in the wavelength of 785 nm with different SiNRs’ gap. (**a**) 30 nm, (**b**) 50 nm, (**c**) 70 nm and (**d**) 90 nm.

Additionally, the influence of employing SiNRs with a different gap on the electric field intensity is also thoroughly investigated, as it is divulged in Figure 2. More specifically, Figure 2a–d depict that the electric field distribution is not decreasing proportionally as the gap of SiNRs elevates from 30 nm–90 nm, whereas the strongest electric field intensity is recorded at a gap value of 50 nm. As shown in Figure 2b, the electrical field intensity around Au nanoparticles is significantly enhanced by localized surface plasmon resonances (LSPR), as well as the large space by LSPR coupling of Au nanoparticles—Au nanoparticles and Au nanoparticles—SiNRs [38]. In general, the coupling of Au nanoparticles is induced by LSPR so that the enhanced electric field on the surface of SiNRs is broadened. Notably, the electrical field intensity at a gap value of 30 nm is even more pronounced, while the light is mainly focused on the top of SiNRs (Figure 2a). As a result, it is anticipated to extremely reduce the SERS enhancement due to the adsorption of a large number of analyte molecules on the bottom of the SiNRs hotspots. Similar to the gap of 30 nm, the weak enhancement of electric field intensity at the gap values of 70 nm and 90 nm is also imposed by the light irradiation on the top of the SiNRs (Figure 2c,d). Therefore, by evaluating the simulation results, a SiNRs’ structure with a length of 700 nm and a gap of 50 nm was designed in order to obtain high sensitivity and uniformity of the SERS substrate.

**Figure 3:**
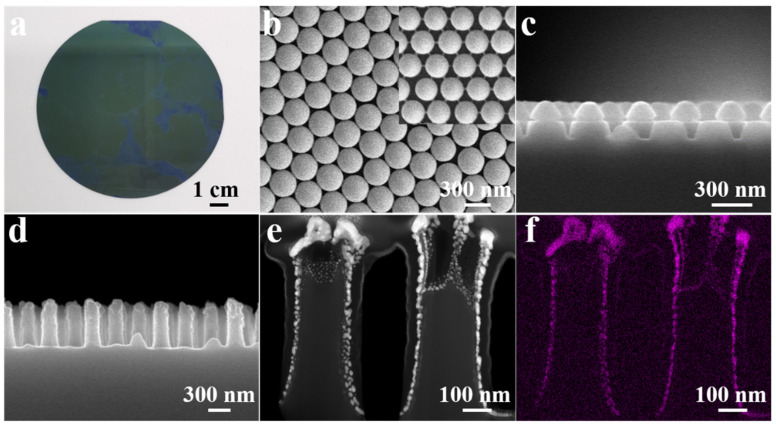
(**a**) The digital image of PS spheres’ monolayer in Si wafer with a diameter of about 10 cm. The SEM images of (**b**) hexagonally packed PS spheres monolayer; the insert is the etched PS spheres’ monolayer, (**c**,**d**) side−view of the SiO_2_ nanorod arrays and the SiNRs. The TEM image of (**e**) the SiNRs decorated with Au nanoparticles and (**f**) mapping of Au element.

According to the above-mentioned results, a SiNRs’ structure with a length of 700 nm and a gap of 50 nm was prepared by combining the self-assembly and the microelectronics manufacturing processes. Firstly, the nano-sized PS spheres’ mask was assembled based on the gas–liquid interface method that was mentioned in our previous work [37]. As it is revealed in the digital image in Figure 3a, the Si wafer with a diameter of about 10 cm was almost completely covered by the PS spheres’ monolayer. Some defects of the PS spheres’ monolayer could be ascribed to the influence of the difference in interfacial tension and the water flow rate on the monolayer transfer process [39]. Consequently, in order to obtain the SiNRs’ structure with a gap value of 50 nm, a hexagonal ordered close-packed PS spheres mask (Figure 3b) was etched by using the ICP technique, where the presence of the reactive O_2_ reduced the diameter of PS spheres from 300 nm to 250 nm in 80 s, as demonstrated in the insert of Figure 3b.

It is interesting to notice that the PS spheres can be easier melted and connected due to the development of a relatively high temperature of ~200 °C during the application of the etching process on the Si substrate. As a result, the employed nano-mask can be destroyed. For that reason, a time-sharing etching and 100 nm SiO_2_ as a dielectric layer were introduced in order to overcome the poor corrosion resistance of the PS spheres and obtain the SiNRs’ structures high aspect ratio. Therefore, by controlling the etching time of 180 s for 10 times, with a power of 300 W, total pressure of 1 pa and CF_4_ flow rate of 40 sccm parameters, SiO_2_ nanorod arrays with a length of 100 nm were fabricated, as it is highlighted in Figure 3c. Moreover, SiNRs with a length of 600 nm were prepared by using a 100 nm SiO_2_ array as a mask at the following condition of time of 420 s, with a power of 300 W, pressure of 5 Pa and an SF_6_ and O_2_ flow of 60 sccm and 10 sccm, respectively (Figure 3d). We have to underline that Au nanoparticles and clusters are favorable for the enhancement of the Raman signal [40]. Consequently, as was described above, the simulations also illustrated that the electrical field intensity around the Au nanoparticles was greatly enhanced by the LSPR effect. Therefore, the SERS substrates were fabricated by depositing 20 nm Au through the sputtering technique and forming Au nanoparticles or clusters on the SiNRs’ structure, as can be observed in Figure 3e,f. Herein, the formation of Au nanoparticles or clusters is ascribed to the conditions of low power, high pressure and short deposition time, under which they can reduce the energy of sputtering clusters to the substrate, migration distance of the clusters on the substrate surface and the total amount of atoms, so that they become fine and dispersed metal particles.

**Figure 4:**
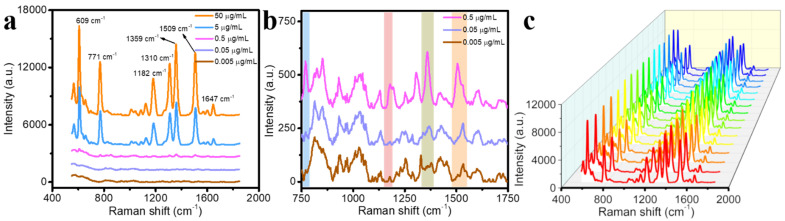
Raman performance of the SERS substrates under the 785 nm wavelength. (**a**) Raman signals of the R6G molecules with concentration value from 5 ng/mL to 50 µg/mL, (**b**) magnified Raman spectra of the R6G molecules from 5 ng/mL to 500 ng/mL and (**c**) sixteen Raman spectra of the R6G molecules with a concentration value of 50 μg/mL.

With the aim to preliminarily verify the SERS activity of the above-mentioned SERS substrate, the sensitivity and reproducibility of the R6G molecules on the SERS substrate were evaluated, and the corresponding SERS spectra are shown in Figure 4. It can be observed that the Raman signal is accordingly diminishing when the R6G solution is diluted from 50 μg/mL to 5 ng/mL, and even when the concentration reaches 5 ng/mL, the Raman spectra can still be clearly identified (Figure 4a,b). Thereafter, experimental data of the R6G fingerprint peak intensity at 1359 cm^−1^, and the molecule concentration are used to access the analytical relationship between them. As it is plotted in Figure S2, a linear relationship can be obtained at a concentration ranging from 50 ng/mL–50 μg/mL. Simultaneously, the SERS reproducibility of R6G at a concentration of 50 μg/mL on the 5 × 5 mm^2^ portions of the SERS substrate was collected as presented in Figure 4c. The small RSD value of only 3.22–4.92% was finally obtained due to the slight fluctuation of the SERS signals. The resulting evidence of the excellent sensitivity and uniformity are ascribed to the ordered Si nanoarrays’ structure and significant Au hotspots.

**Figure 5:**
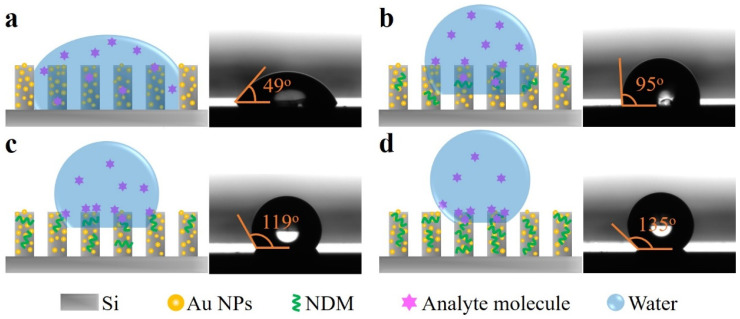
Schematic illustration of droplet contact behaviors on the SERS substrates soaked in NDM solution with different time and contact angles: (**a**) 0 s, (**b**) 60 s, (**c**) 300 s and (**d**) 900 s.

In order to better understand the impact of the wettability on the detection and analysis properties of the SERS substrate, different wettability SERS substrates were prepared by soaking the initially obtained SERS substrate into the NDM solution for 60 s, 300 s, and 900 s, respectively. The results are illustrated in Figure 5a, where we can observe that the SERS substrate surface became hydrophilic after the Au sputtering process due to its relatively high surface energy and as a result displayed a contact angle of 49°. Consequently, the analyte molecules are diluted to their concentration and uniformly dispersed on a large area of SiNRs. In Figure 5b–d, the SERS substrate surface exhibit a hydrophobicity with contact angles of 95°, 119° and 135°, respectively, which is ascribed to the strong interaction between sulfhydryl and Au and the introduced carbon chain during soaking in NDM. After the droplet evaporation on the hydrophobic SERS substrate, the molecules are enriched in small spots. Meanwhile, as can be observed from Figure 5c, when the SERS substrate was immersed within NDM for a total period of 300 s with a contact angle of 119°, the analyte molecules were almost completely located at the top area of the SiNR as the droplets evaporated. Hence, an extremely efficient attachment of the molecules was achieved. Furthermore, as it is divulged in Figure 5d, the droplet contact angle reached the value of 135° in order to attain the superhydrophobicity properties, and the analyte molecules were further accumulated and dispersed at an even smaller optimal hotspots area after the droplet evaporation. There is a stronger Raman signal of analyte molecules on hydrophobic substrate than those on hydrophilic substrate even for the same concentration of analyte molecules in the theory. However, as a result, some challenges and difficulties of analyte molecules’ adhesion to the substrate, the coverage of Au nanoparticles hotspots by excess NDM molecules, and caused microstructure damage with a prolonged immersion time, are introduced.

**Figure 6:**
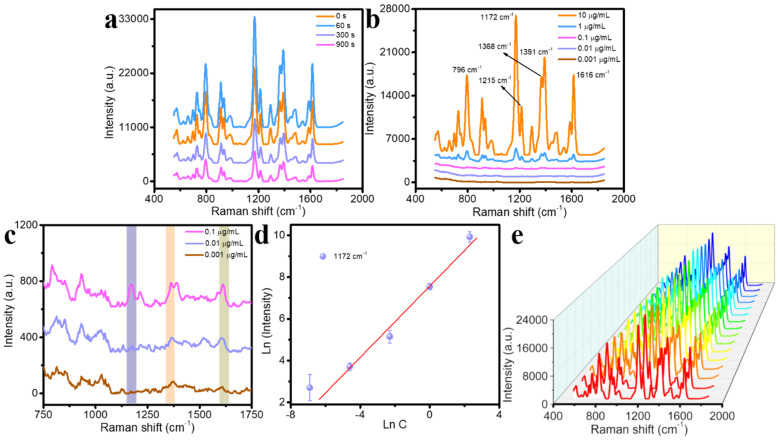
Raman spectra of the hydrophobic SERS substrates under the 785 nm wavelength. (**a**) Raman signals of the MG molecules with a concentration value of 10 μg/mL on the SERS substrates with different wettabilities. Raman performance of the MG molecules on the SERS substrates were immersed within NDM for 60 s: (**b**) concentration of the MG molecules from 1 ng/mL to 10 μg/mL, (**c**) magnified Raman spectra of the MG molecules from 1 ng/mL to 100 ng/mL, (**d**) the logarithmic function between Raman intensity of Raman band at 1172 cm^−1^ and the MG concentration ranging from 1 ng/mL to 10 μg/mL, and (**e**) sixteen Raman spectra of the MG molecules with a concentration value of 10 μg/mL.

In order to gain more insight and explore a practical monitoring of trace pesticide residues, malachite green, which is a kind of fungicide commonly found in fish products, is employed as a probe molecule for verifying the sensing activity of the prepared SERS substrates. The SERS measurements of MG with a concentration value of 10 μg/mL on different wettability SERS substrates at the excitation wavelengths of 785 nm were conducted, as it is revealed in Figure 6a. More specifically, when the soaking time of the SERS substrate was less than 60 s, the acquired Raman signals of MG gradually enhanced as the hydrophobicity increased. Nevertheless, by increasing the soaked time from 60 s to 900 s, the Raman signals became slightly shrunk. This effect indicates that the additional soaking time does not significantly contribute to the SERS activity but results in a larger droplet contact angle, thus reducing the contact area between the analyte molecules and the hotspots. Meanwhile, combining Figure 6a and Figure S3, the Raman intensity value of ~1.5 × 10^4^ a.u. induced from the plasma resonance enhancement imposed by Au nanoparticles for the 1172 cm^−1^ band of MG with a concentration value of 10 μg/mL on original substrate was increased to ~2.3 × 10^4^ a.u. for modified SERS substrate within NDM for 60 s, and a difference of ~0.8 × 10^4^ a.u. was collected. About 53.3% of the Raman intensity increment can be evaluated resulting from a chemical enhancement molecule enrichment due to the SERS substrate hydrophobicity. Consequently, the SERS substrate with a soaking time within NDM of 60 s was selected in order to characterize and analyze the ability for detection of the pesticide residues of MG. Besides, in order to exclude Raman signals’ interference of NDM molecules absorbed on the SERS substrate, the SERS signals of the substrates initially and after immersion in the NDM solution for 60 s were measured, as displayed in Figure S4. It can be found that there are no obvious Raman characteristic peaks are observed except for the weak background signals, indicating that it has no effect of NDM molecules absorbed on the SERS substrate on the pesticide residue of the MG measurement.

It is interesting to notice that Figure 6b,c reveal the Raman intensity of MG by varying the employed concentrations from the value of 1 ng/mL to 10 μg/mL at an excitation wavelength of 785 nm in order to further examine the sensitivity of the SERS substrate. As can be ascertained from Figure 6b, the MG compound possesses discernible Raman characteristic peaks of 796 cm^−1^, 1172 cm^−1^, 1215 cm^−1^, 1368 cm^−1^, 1391 cm^−1^ and 1616 cm^−1^ at the concentration values of 10 μg/mL and 1 μg/mL. Besides, in Figure 6c, the respective Raman signal of MG with a concentration of 1 ng/mL can be also detected, signifying that the proposed configuration presents a low detection limit. The excellent sensitivity is induced as a combination of the plasma resonance enhancement imposed by Au nanoparticles and the molecule enrichment from the SERS substrate hydrophobic ability. On top of that, Figure 6d reveals a well defined logarithmic relationship between the Raman intensity of the characteristic peaks at 1172 cm^−1^ and the MG concentration ranging from 10 ng/mL to 10 μg/mL, which confirms that the SERS substrate has an outstanding quantitative analysis capability at a concentration greater than 10 ng/mL in theory. Whereas, it is clear that Raman signal intensity has deviated from the linear equation at a concentration of 1 ng/mL, indicating that the prepared SERS substrate still faces great difficulties in detecting target molecules of low-concentration.

Furthermore, to examine the reproducibility of the SERS performance on hydrophobic SiNRs decorated by 20 nm Au nanoparticles, the SERS spectra of 10 μg/mL MG were measured on 16 random positions of the same SERS substrate and the SERS platforms of 10, depicted in Figure 6e and Figure S5. The obtained RSD reached as low as 4.04–6.14% (Table 1) for the same SERS substrate and an RSD of 8.33–12.09% was obtained based on 10 SERS platforms, respectively, which was lower than that in the literature [41,42] and exhibited the outstanding reproducibility of the employed SERS substrate. This great reproducibility is attributed to the ordered distribution of the SiNRs’ structure and the Au nanoparticles, which provide homogeneous, symmetrical and substantial electromagnetic hotspots. Additionally, the enhancement factor (EF) as an important parameter was employed to evaluate the enhancement effect of the SERS substrate and calculated as disclosed in the Supplementary Materials. Based on Figure S6, it was noted that an EF value of above 4.0 × 10^6^ for a 1172 cm^−1^ band of MG molecules on the hydrophobic SERS substrate was obtained relative to the planar Si wafer. Meanwhile, by comparing with the EF values and detection limits in the latest literature and this work (Table S1), it was found that one of the parameters in the literature was excellent, but both parameters were ideal in our work, which suggested that the SERS substrate mentioned in this work had the potential to be applied in the trace detection of pesticide residue.

## 4. Conclusions

In summary, a prominent SERS substrate was demonstrated based on SiNRs’ structure decorated with Au nanoparticles and modified with NDM. The proposed configuration exhibits the features of hydrophobicity and wafer-scale uniformity. The fabricated SERS substrate not only possessed the advantage of the strong localized surface plasmon resonance effect induced by the presence of Au nanoparticles, but also provided a unique pathway for the molecule enrichment resulting from the surface hydrophobic modification. The SERS substrate was consequently used for the detection of the pesticide residue of MG, with a detection limit of 1 ng/mL with a RSD as low as 4.04–6.14%. Moreover, the extracted EF reached the value of 10^6^, whereas a good linear relationship between the logarithmic Raman intensity and the MG concentration was achieved, as the latter varied from 10 ng/mL to 10 μg/mL. As a result, the high SERS sensitivity, reproducibility and quantitative nature of the employed SERS substrate render it suitable for practical sensing applications.

## Figures and Tables

**Table 1:** The RSD value of MG molecules’ Raman peaks from 16 Raman spectra obtained on the hydrophobic SERS substrate.

Raman Shift	796 cm^−1^	1172 cm^−1^	1215 cm^−1^	1368 cm^−1^	1391 cm^−1^	1616 cm^−1^
RSD	6.14%	4.04%	6.08%	4.51%	4.24%	5.51%

## Data Availability

Not applicable.

## Supplementary Materials

The following supporting information can be downloaded at: https://www.mdpi.com/article/10.3390/bios12050273/s1, Figure S1: The simulated model with Au nanoparticles on Si nanorods, Figure S2: The logarithmic function between Raman intensity of Raman band at 1359 cm−1 and the R6G concentration ranging from 5 ng/mL to 50 μg/mL, Figure S3: Raman spectra of MG molecules on the original SERS substrates under the 785 nm wavelength. (a) Concentration of MG from 1 ng/mL to 10 μg/mL, and (b) magnified Raman spectra of MG from 1 ng/mL to 100 ng/mL, Figure S4: The SERS signals of the substrates initially and after immersion in NDM solution for 60 s, Figure S5: Raman spectra of MG molecules with a concentration value of 10 μg/mL on 10 SERS platforms, Figure S6: Raman spectra of 10 μg/mL MG adsorbed on the SERS substrate and 100 mg/mL MG adsorbed on planar Si wafer, Table S1: Comparison of EF values and limitations of detection for MG on the SERS substrates in this work and the latest literature.

## References

[1] Peng Y., Lin C., Li Y., Gao Y., Wang J., He J., Huang Z., Liu J., Luo X., Yang Y. (2022). Identifying infectiousness of SARS-CoV-2 by ultra-sensitive SnS2 SERS biosensors with capillary effect. Matter.

[2] Han C., Li W., Li Q., Xing W., Luo H., Ji H., Fang X., Luo Z., Zhang L. (2022). CRISPR/Cas12a-Derived electrochemical aptasensor for ultrasensitive detection of COVID-19 nucleocapsid protein. Biosens. Bioelectron..

[3] Poore G.D., Kopylova E., Zhu Q., Carpenter C., Fraraccio S., Wandro S., Kosciolek T., Janssen S., Metcalf J., Song S.J. (2020). Microbiome analyses of blood and tissues suggest cancer diagnostic approach. Nature.

[4] Leong S.X., Leong Y.X., Tan E.X., Sim H.Y.F., Koh C.S.L., Lee Y.H., Chong C., Ng L.S., Chen J.R.T., Pang D.W.C. (2022). Noninvasive and Point-of-Care Surface-Enhanced Raman Scattering (SERS)-Based Breathalyzer for Mass Screening of Coronavirus Disease 2019 (COVID-19) under 5 min. ACS Nano.

[5] Nowak M., Trojanowska A., Marciniak L., Binczyk M., Runka T., Tylkowski B., Jastrzab R. (2019). Preparation and characterization of long-term stable SERS active materials as potential supports for medical diagnostic. Appl. Surf. Sci..

[6] Ben-Jaber S., Peveler W.J., Quesada-Cabrera R., Cortes E. (2016). Sotelo-Vazquez, C.; Abdul-Karim, N.; Maier, S.A.; Parkin, I.P., Photo-induced enhanced Raman spectroscopy for universal ultra-trace detection of explosives, pollutants and biomolecules. Nat. Commun..

[7] Krivitsky V., Filanovsky B., Naddaka V., Patolsky F. (2019). Direct and Selective Electrochemical Vapor Trace Detection of Organic Peroxide Explosives via Surface Decoration. Anal. Chem..

[8] Dincer C., Bruch R., Costa-Rama E., Fernandez-Abedul M.T., Merkoci A., Manz A., Urban G.A., Guder F. (2019). Disposable Sensors in Diagnostics, Food, and Environmental Monitoring. Adv. Mater..

[9] Yu Z., Jung D., Park S., Hu Y., Huang K., Rasco B.A., Wang S., Ronholm J., Lu X., Chen J. (2022). Smart traceability for food safety. Crit. Rev. Food Sci. Nutr..

[10] Zong C., Xu M., Xu L.J., Wei T., Ma X., Zheng X.S., Hu R., Ren B. (2018). Surface-Enhanced Raman Spectroscopy for Bioanalysis: Reliability and Challenges. Chem. Rev..

[11] Zhan C., Chen X.-J., Yi J., Li J.-F., Wu D.-Y., Tian Z.-Q. (2018). From plasmon-enhanced molecular spectroscopy to plasmon-mediated chemical reactions. Nat. Rev. Chem..

[12] Huang Y., Wang X., Lai K., Fan Y., Rasco B.A. (2020). Trace analysis of organic compounds in foods with surface-enhanced Raman spectroscopy: Methodology, progress, and challenges. Compr. Rev. Food Sci. Food Saf..

[13] Ding S.Y., You E.M., Tian Z.Q., Moskovits M. (2017). Electromagnetic theories of surface-enhanced Raman spectroscopy. Chem. Soc. Rev..

[14] Yang Z.W., Meng L.Y., Lin J.S., Yang W.M., Radjenovic P., Shen S.X., Xu Q.C., Yang Z.L., Tian Z.Q., Li J.F. (2019). 3D Hotspots Platform for Plasmon Enhanced Raman and Second Harmonic Generation Spectroscopies and Quantitative Analysis. Adv. Opt. Mater..

[15] Ding S.-Y., Yi J., Li J.-F., Ren B., Wu D.-Y., Panneerselvam R., Tian Z.-Q. (2016). Nanostructure-based plasmon-enhanced Raman spectroscopy for surface analysis of materials. Nat. Rev. Mater..

[16] Wang X., Guo L. (2020). SERS Activity of Semiconductors: Crystalline and Amorphous Nanomaterials. Angew. Chem. Int. Ed. Engl..

[17] Ran P., Jiang L., Li X., Li B., Zuo P., Lu Y. (2019). Femtosecond Photon-Mediated Plasma Enhances Photosynthesis of Plasmonic Nanostructures and Their SERS Applications. Small.

[18] Chen Y., Liu H., Tian Y., Du Y., Ma Y., Zeng S., Gu C., Jiang T., Zhou J. (2020). In Situ Recyclable Surface-Enhanced Raman Scattering-Based Detection of Multicomponent Pesticide Residues on Fruits and Vegetables by the Flower-like MoS2@Ag Hybrid Substrate. ACS Appl. Mater. Interfaces.

[19] Huang Z., Meng G., Hu X., Pan Q., Huo D., Zhou H., Ke Y., Wu N. (2018). Plasmon-tunable Au@Ag core-shell spiky nanoparticles for surface-enhanced Raman scattering. Nano Res..

[20] Wang C., Xu X., Qiu G., Ye W., Li Y., Harris R.A., Jiang C. (2021). Group-Targeting SERS Screening of Total Benzodiazepines Based on Large-Size (111) Faceted Silver Nanosheets Decorated with Zinc Oxide Nanoparticles. Anal. Chem..

[21] Liu C., Yang M., Yu J., Lei F., Wei Y., Peng Q., Li C., Li Z., Zhang C., Man B. (2020). Fast multiphase analysis: Self-separation of mixed solution by a wettability-controlled CuO@Ag SERS substrate and its applications in pollutant detection. Sens. Actuators B Chem..

[22] Guang Y., Peng Y., Yan Z., Liu Y., Zhang J., Zeng X., Zhang S., Zhang S., Burn D.M., Jaouen N. (2020). Electron Beam Lithography of Magnetic Skyrmions. Adv. Mater..

[23] Probst C., Meichner C., Kreger K., Kador L., Neuber C., Schmidt H.W. (2016). Athermal Azobenzene-Based Nanoimprint Lithography. Adv. Mater..

[24] Politano G.G., Cazzanelli E., Versace C., Vena C., De Santo M.P., Castriota M., Ciuchi F., Bartolino R. (2018). Graphene oxide on magnetron sputtered silver thin films for SERS and metamaterial applications. Appl. Surf. Sci..

[25] Demirel G., Gieseking R.L.M., Ozdemir R., Kahmann S., Loi M.A., Schatz G.C., Facchetti A., Usta H. (2019). Molecular engineering of organic semiconductors enables noble metal-comparable SERS enhancement and sensitivity. Nat. Commun..

[26] Tian Z.Q., Ren B., Li J.F., Yang Z.L. (2007). Expanding generality of surface-enhanced Raman spectroscopy with borrowing SERS activity strategy. Chem. Commun. (Camb).

[27] Yu J., Yang M., Li Z., Liu C., Wei Y., Zhang C., Man B., Lei F. (2020). Hierarchical Particle-In-Quasicavity Architecture for Ultratrace In Situ Raman Sensing and Its Application in Real-Time Monitoring of Toxic Pollutants. Anal. Chem..

[28] Qi Q., Liu C., Liu L., Meng Q., Wei S., Ming A., Zhang J., Wang Y., Wu L., Zhu X. (2019). Fabrication, Characterization, and Application of Large-Scale Uniformly Hybrid Nanoparticle-Enhanced Raman Spectroscopy Substrates. Micromachines.

[29] Li P., Cao M., Bai H., Zhao T., Ning Y., Wang X., Liu K., Jiang L. (2019). Unidirectional Liquid Manipulation Via an Integrated Mesh with Orthogonal Anisotropic Slippery Tracks. Adv. Funct. Mater..

[30] Niu Z.Z., Gao F.Y., Zhang X.L., Yang P.P., Liu R., Chi L.P., Wu Z.Z., Qin S., Yu X., Gao M.R. (2021). Hierarchical Copper with Inherent Hydrophobicity Mitigates Electrode Flooding for High-Rate CO2 Electroreduction to Multicarbon Products. J. Am. Chem. Soc..

[31] Li F., Wu W., Wang S. (2021). Pore network simulations of liquid water and oxygen transport in gas diffusion layers with spatially variable wettability. J. Power Sources.

[32] Huang C.J., Fang W.F., Ke M.S., Chou H.Y., Yang J.T. (2014). A biocompatible open-surface droplet manipulation platform for detection of multi-nucleotide polymorphism. Lab Chip.

[33] Xu T., Xu L.P., Zhang X., Wang S. (2019). Bioinspired superwettable micropatterns for biosensing. Chem. Soc. Rev..

[34] Zhou B., Mao M., Cao X., Ge M., Tang X., Li S., Lin D., Yang L., Liu J. (2018). Amphiphilic Functionalized Acupuncture Needle as SERS Sensor for In Situ Multiphase Detection. Anal. Chem..

[35] Li R., Mao H., Shi M., Zhao Q., Chen D., Xiong J. (2020). Parahydrophobic 3D nanohybrid substrates with two pathways of molecular enrichment and multilevel plasmon hybridization. Sens. Actuators B Chem..

[36] Li H., Yang Q., Hou J., Li Y., Li M., Song Y. (2018). Bioinspired Micropatterned Superhydrophilic Au-Areoles for Surface-Enhanced Raman Scattering (SERS) Trace Detection. Adv. Funct. Mater..

[37] Chen F., Qi Q., Zhao Y., Zhang S., Zhao Y., Ming A., Mao C. Large-scale Uniformly Hybrid Micro-nano Structure Wetting Solid Substrate for Surface-enhanced Raman Spectroscopy. Proceedings of the 2021 IEEE 16th International Conference on Nano/Micro Engineered and Molecular Systems (NEMS).

[38] Lin D., Wu Z., Li S., Zhao W., Ma C., Wang J., Jiang Z., Zhong Z., Zheng Y., Yang X. (2017). Large-Area Au-Nanoparticle-Functionalized Si Nanorod Arrays for Spatially Uniform Surface-Enhanced Raman Spectroscopy. ACS Nano.

[39] Lotito V., Zambelli T. (2017). Approaches to self-assembly of colloidal monolayers: A guide for nanotechnologists. Adv. Colloid Interface Sci..

[40] Guo Q., Xu M., Yuan Y., Gu R., Yao J. (2016). Self-Assembled Large-Scale Monolayer of Au Nanoparticles at the Air/Water Interface Used as a SERS Substrate. Langmuir.

[41] Luo J., Wang Z., Li Y., Wang C., Sun J., Ye W., Wang X., Shao B. (2021). Durable and flexible Ag-nanowire-embedded PDMS films for the recyclable swabbing detection of malachite green residue in fruits and fingerprints. Sens. Actuators B Chem..

[42] Yang W., Tang J., Ou Q., Yan X., Liu L., Liu Y. (2021). Recyclable Ag-Deposited TiO2 SERS Substrate for Ultrasensitive Malachite Green Detection. ACS Omega.

