# Supplementary material for: Hydrophobic Wafer-Scale High-Reproducibility SERS Sensor Based on Silicon Nanorods Arrays Decorated with Au Nanoparticles for Pesticide Residue Detection
Source: Biosensors (Basel). 2022 Apr 26;12(5):273. doi: 10.3390/bios12050273 (PMC9138717; doi:10.3390/bios12050273)
Supplement: Supplementary file 1 [file biosensors-12-00273-s001.zip › biosensors-1674956-supplementary.pdf]

Supplementary Materials

Hydrophobic Wafer-Scale High-Reproducibility SERS Sensor Based on Silicon Nanorods Arrays Decorated with Au Nanoparticles for Pesticide Residue Detection

Fanhong Chen [1,2], Yupeng Zhao [2,3], Shaoxun Zhang [1,2], Shuhua Wei [3], Anjie Ming [1,2,*] and Changhui Mao [1,2,*]

1 State Key Laboratory of Advanced Materials for Smart Sensing, GRINM Group Corporation Limited, Beijing 100088, China; chenfanhong@grinm.com (F.C.); 17863137556@163.com (S.Z.)

2 Department of Advanced Electronic Materials, GRIMAT Engineering Institute Co., Ltd., Beijing 101407, China; zhaoyp@mail.ncut.edu.cn

3 School of Information Science and Technology, North China University of Technology, Beijing 100144, China; weishuhua@ncut.edu.cn

* Correspondence: mingaj@grinm.com (A.M.); mao@grinm.com (C.M.)

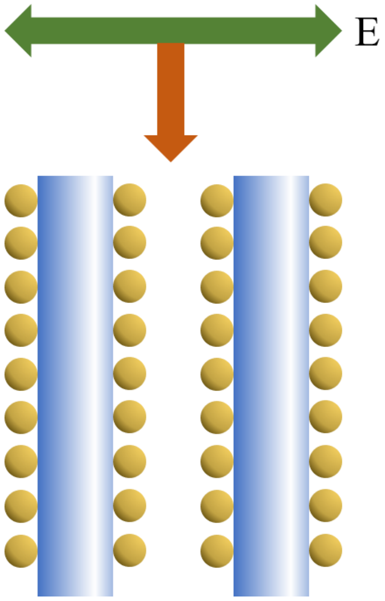

Figure S1. The simulated model with Au nanoparticles on Si nanorods.

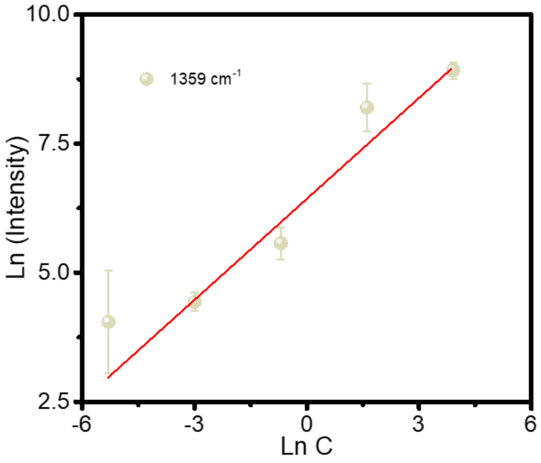

Figure S2. The logarithmic function between Raman intensity of Raman band at 1359 cm^{-1} and the R6G concentration ranging from 5 ng/mL to 50 μg/mL.

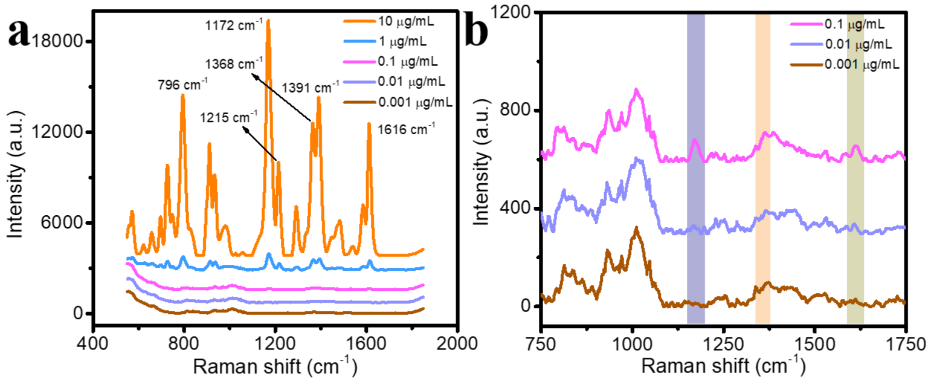

Figure S3. Raman spectra of MG molecules on the original SERS substrates under the 785 nm wavelength. **(a)** concentration of MG from 1 ng/mL to 10 μg/mL, and **(b)** magnified Raman

spectra of MG from 1 ng/mL to 100 ng/mL.

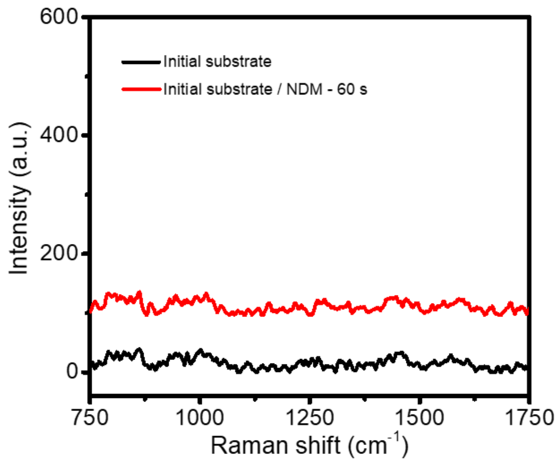

Figure S4. The SERS signals of the substrates initially and after immersion in NDM solution for 60 s.

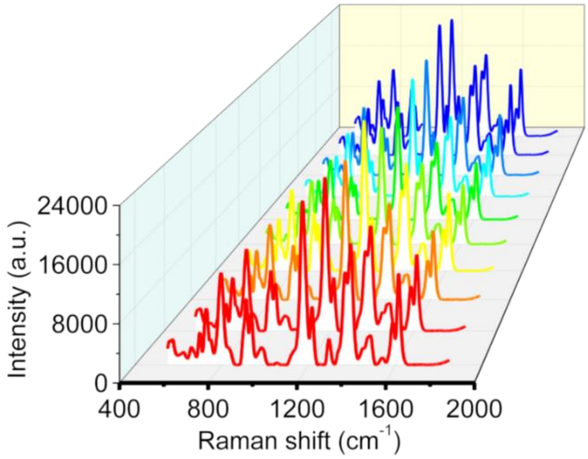

Figure S5. Raman spectra of MG molecules with a concentration value of 10 μg/mL on 10 SERS platforms.

The EF value of the SERS substrate was calculated by using the following Equation S1 [*Nano-Micro Lett.* **2021**, 13(109)],

$$EF = \frac{I_{SERS}}{I_{REF}} \times \frac{N_{REF}}{N_{SERS}} \tag{S1}$$

where I_{SERS} and I_{REF} are Raman intensities of the adsorbed MG molecules on the SERS substrate and planar Si wafer, respectively. N_{SERS} and N_{REF} are the total number probed on the SERS substrate and the average number excited by applying the laser

irradiation on planar Si wafer for MG molecules. As for the numerator ratio of molecules in a Raman focused window on the SERS and silicon substrates is shown in follows:

$$\frac{S_{SERS}}{S_{REF}} = \frac{\pi \times 0.5^2 + 2 \times \pi \times 0.125 \times 0.7 \times 11 \times \pi \times 0.5^2}{\pi \times 0.5^2} \quad \text{(S2)}$$

in this formula, the diameter and length of the nanorods are around 250 nm and 700 nm, and the density of nanorods is approximately 11 μm^{-2}. The diameter of the focused area for Raman measurements is 1 μm. The number of molecules in a Raman focused area on the SERS substrate was derived from Equation S2 was 7 times that of planar Si wafer for the same concentration of probe molecule solution.

$$\frac{N_{REF}}{N_{SERS}} = 1.4 \times 10^3 \quad \text{(S3)}$$

Therefore, the ratio value of N_{REF} and N_{SERS} was obtained for 10 μg/mL MG absorbed on the SERS substrate and 100 mg/mL MG on planar Si wafer as shown in Equation S3. Then combining Equations S1, S3 and Figure S6, an EF value of above 4.0×10^6 for 1172 cm^{-1} band of MG molecules on the hydrophobic SERS substrate was obtained relative to planar Si wafer.

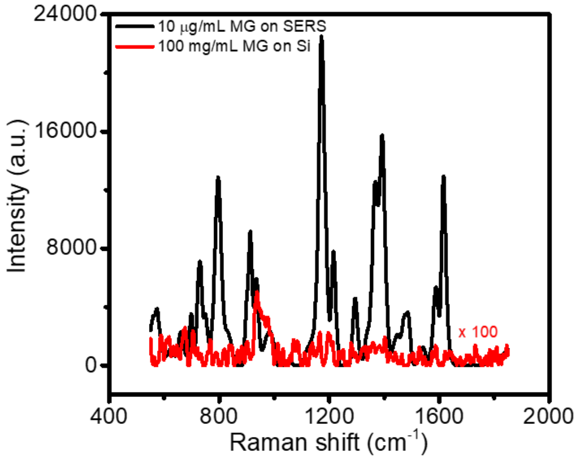

Figure S6. Raman spectra of 10 μg/mL MG adsorbed on the SERS substrate and 100 mg/mL MG adsorbed on planar Si wafer.

Table S1. Comparison of EF values and limitations of detection for MG on the SERS substrates in this work and latest literatures.

EF	detection limit	SERS substrate	reference
~10^6	~10^{-9} M	SiNRs/Au	this work
~10^8		Ag nanorods	*Appl. Phys. Lett.* **2005**, 87, 031908
~10^6	~10^{-9} M	Au@SiO_2 NPs	*Vib. Spectrosc.* **2022**, 118, 103319
	~10^{-15} M	Au/Ag nanocone	*Sensors & Actuators B: Chemical* **2020**, 320
	~10^{-10} M	Ag needle@Au	*ACS Appl. Nano Mater.* **2019**, 2, 5
	~10^{-16} M	TiO_2/Ag	*ACS Omega* **2021**, 6, 27271-27278
